# The Regulatory Network of Gastric Cancer Pathogenesis and Its Potential Therapeutic Active Ingredients of Traditional Chinese Medicine Based on Bioinformatics, Molecular Docking, and Molecular Dynamics Simulation

**DOI:** 10.1155/2022/5005498

**Published:** 2022-11-26

**Authors:** Peng Yang, Peng Liu, Junmao Li

**Affiliations:** ^1^Ganzhou People's Hospital/Ganzhou Hospital Affiliated to Nanfang Hospital, Southern Medical University, Ganzhou 341000, China; ^2^The National Pharmaceutical Engineering Center for Solid Preparationin Chinese Herbal Medicine, Jiangxi University of Chinese Medicine, Jiangxi, Nanchang 330006, China

## Abstract

**Objective:**

This study aims to investigate the functional gene network in gastric carcinogenesis by using bioinformatics; besides, the diagnostic utility of key genes and potential active ingredients of traditional Chinese medicine (TCM) for treatment in gastric cancer have been explored.

**Methods:**

The Cancer Genome Atlas and Gene Expression Omnibus databases have been applied to analyze the differentially expressed genes (DEGs) between gastric cancer and normal gastric tissues. Then, the DEGs underwent Gene Ontology and Kyoto Encyclopedia of Genes and Genomes enrichment analyses using the Metascape database. The STRING database and the Cytoscape software were utilized for the protein-protein interaction network of DEGs and hub genes screening. Furthermore, survival and expression analyses of hub genes were conducted using Gene Expression Profiling Interactive Analysis and Human Protein Atlas databases. By using the Comparative Toxicogenomics Database, the hub genes interconnected with active ingredients of TCM were analyzed to provide potential information for the treatment of gastric cancer. After the molecular docking of the active ingredients of TCM to specific hub gene receptor proteins, the molecular dynamics simulation GROMACS was applied to validate the conformation of the strongest binding ability in the molecular docking.

**Results:**

A total of 291 significant DEGs were found, from which 12 hub genes were screened out. Among these hub genes, the expressions of five hub genes including COL1A1, COL5A2, MMP12, SERPINE1, and VCAN were significantly correlated with the overall survival. Furthermore, four potential therapeutic active ingredients of TCM were acquired, including quercetin, resveratrol, emodin, and schizandrin B. In addition, the molecular docking results exhibited that the active ingredients of TCM formed stable binding with the hub gene targets. SERPINE1 (3UT3)-Emodin and COL1A1 (7DV6)-Quercetin were subjected to molecular dynamics simulations as conformations of continuing research significance, and both were found to be stably bound as a result of the interaction of van der Waals potentials, electrostatic, and hydrogen bonding.

**Conclusion:**

Our findings may provide novel insights and references for the screening of biomarkers, the prognostic evaluation, and the identification of potential active ingredients of TCM for gastric cancer treatment.

## 1. Introduction

Gastric cancer, as a malignancy occurring in the gastric mucosal epithelium, is the fifth most frequent cancer and the third most common cause of cancer deaths worldwide [1], with 1.48 million new cases annually. The occurrence and development of gastric cancer is a complicated process involving multiple factors, steps, and genes, whilst the etiology and pathogenesis have not been fully elucidated so far. It is well accepted that the risk factors of gastric cancer contain diet, lifestyle (smoking and alcohol consumption), and *Helicobacter pylori* infection [2]. The main therapeutic approaches for gastric cancer in recent years were endoscopic resection [3], surgery [4], radiotherapy [5], neoadjuvant chemotherapy [6], immunotherapy [7], and traditional Chinese medicine (TCM) [8]. Early gastric cancer is difficult to be diagnosed due to its insidious onset, resulting in a large number of patients missing the golden treatment period and even approximately 70% of patients with gastric cancer at an advanced stage of diagnosis [9], which severely limits the efficacy of surgery and radiotherapy [9]. Although conventional chemotherapeutic agents, such as cisplatin and 5-fluorouracil, have provided enormous clinical benefits for patients with advanced gastric cancer, they pose a huge challenge to treatment because of their resistance and cytotoxicity [10, 11]. Therefore, the search for genes tightly related to the development of gastric cancer is highly valuable for clarifying the pathogenesis of gastric cancer and dissecting potential drugs to prevent and treat gastric cancer.

TCM has historically been known for its multitargeting and low adverse effects, which has great advantages in improving the quality of life of patients with digestive system diseases [12, 13]. Of note, a large number of studies have reported that TCM combined with chemotherapeutic drugs improves the survival and prognosis of patients with gastric cancer [14–16]. Mechanistic research [16] has manifested that TCM and its active ingredients can exert antitumor effects through various mechanisms, such as inhibition of cell proliferation, interference with angiogenesis, repression of cell motility, and regulation of inflammation-related factors. Therefore, it is evident that the antigastric cancer effects of TCM and its active ingredients cannot be underestimated. This study set out to dissect the hub genes affecting gastric carcinogenesis by mining diverse bioinformatics databases and to search for potential therapeutic targets to provide a bioinformatics basis for the discovery of active ingredients of TCM, which were finally confirmed by molecular docking and molecular dynamics simulation techniques.

## 2. Materials and Methods

### 2.1. Data Collection

The transcriptome data of the tumor tissues of 375 patients with gastric cancer and 32 matched normal tissues were downloaded from The Cancer Genome Atlas (TCGA) database (https://cancergenome.nih.gov, it was accessed on November 15, 2021). The Gene Expression Omnibus (GEO) database (https://www.ncbi.nlm.nih.gov/gds/, it was accessed on November 20, 2021) was utilized to find datasets (GSE103236 and GSE54129), which matched the requirements of this study, of differentially expressed gene (DEG) profiles between gastric cancer and adjacent normal tissues that with gastric cancer as the keyword. Among them, the GSE103236 microarray data contained 10 gastric cancer samples and 9 normal tissue samples, and the GSE54129 microarray data consisted of 111 gastric cancer samples and 21 normal tissue samples.

### 2.2. Experimental Methods

#### 2.2.1. Screening of DEGs between Gastric Cancer and Adjacent Normal Tissues

DEGs in gastric cancer were screened in the transcriptome data of tumor tissues of patients with gastric cancer and 32 matching normal tissues downloaded from the TCGA database using the R language software with |log2 fold change (log2FC)| ≥ 2 and false discovery rate (FDR) < 0.01 as screening criteria. The expression matrices of gastric cancer and normal tissues in GSE103236 and GSE54129 microarray samples were differentially analyzed by GEO 2R with the screening criteria of |log2FC| ≥ 1 and FDR < 0.05, respectively, followed by the comparison of the DEG datasets between gastric cancer and normal groups in the two sets of microarray data. Then, the combination was conducted on the DEGs obtained from the data of the two microarray samples. Finally, the DEGs harvested from TCGA were intersected with the DEGs attained from the 2 sets of GEO microarray samples, followed by the plotting of the Veen diagram to acquire the intersecting genes, that is, gastric cancer-related DEGs.

#### 2.2.2. Gene Ontology (GO) and Kyoto Encyclopedia of Genes and Genomes (KEGG) Pathway Enrichment Analyses

Metascape (https://metascape.org/, it was visited on November 25, 2021) is a repository that annotates the biological functions of genes and proteomes. In this study, the Metascape database was employed to perform the GO and KEGG enrichment analyses on the obtained DEGs. In addition, *P* < 0.05 was adopted as the basis for determining the specificity of significant DEGs in the pathway enrichment analysis, biological process (BP), molecular function (MF), and cell components (CC).

#### 2.2.3. Construction of the Protein-Protein Interaction (PPI) Network and Acquisition of Hub Genes

The intersecting genes were imported into the STRING database (https://stringdb.org/, which was visited on December 5, 2021). The PPI network of significant DEGs was constructed by setting the interaction score threshold to >0.400, limiting the study population to human species, and hiding the free genes disconnected from the networks, followed by the exportation of the string-interactions file. The string-interactions file was imported into the Cytoscape software. Afterward, the PPI network was scored twice using the CytoNCA plug-in for betweenness (BC), closeness (CC), degree (DC), eigenvector (EC), local average connectivity-based method (LAC), network (NC), subgraph (SC), and information (IC). The hub genes of the PPI network were retrieved based on the genes with scores greater than the median value.

#### 2.2.4. Survival Analysis and Validation of Hub Genes

The Gene Expression Profiling Interactive Analysis (GEPIA) database (https://gepia.cancer-pku.cn, it was accessed on December 15, 2021) [17] is a database containing gene expression profiles of various tumors and cancers, which can be utilized to assess the mRNA expression of genes in the prognosis of gastric cancer and explore the impact of high and low expression of genes on the overall survival of patients with gastric cancer. The hub genes obtained from 1.2.3 were sequentially imported into the GEPIA database. The patients in the corresponding dataset were arranged into high and low-expression groups as per the median gene expression, followed by statistical analyses using the log-rank test. In the analyses, *P* < 0.05 was considered statistically significant and acted as a basis for identifying whether hub genes were correlated with the prognosis of patients, and the hazard ratios (HR) indicated the probability of cancer progression or death in patients with a high gene expression relative to those with low gene expression. The GEPIA database was adopted to verify the expression of the hub genes in gastric cancer and normal gastric tissues detected by RNA sequencing, with the results shown in box plots.

#### 2.2.5. The Pathology Section Assay of the Hub Genes

The expression of the proteins encoded by the hub genes in gastric cancer and normal gastric tissues was examined using the Human Protein Atlas (HPA) database (https://www.proteinatlas.org, it was accessed on December 25, 2021), with the collection of representative immunohistochemistry staining images.

#### 2.2.6. Screening of Active Ingredients of TCM Targeting Core Pathogenic Genes of Gastric Cancer

The Comparative Toxicogenomics Database (CTD, https://ctdbase.org, it was visited on December 30, 2021), as an innovative digital ecosystem that relates toxicological information for chemicals, genes, phenotypes, diseases, and exposures, can be applied for the research of the interaction between gene targets and active ingredients of TCM [18]. The hub genes were imported into the search box to retrieve the compounds interacting with the hub genes with humans as the species. Thereafter, the compounds were exported in the form of an Excel sheet to analyze the involved active ingredients of TCM using the interaction value >1 as the screening criteria.

#### 2.2.7. Molecular Docking

The structural formulas of active components were downloaded from the PubChem database (https://pubchem.ncbi.nlm.nih.gov/, it was accessed on January 5, 2022). The corresponding three-dimensional (3D) structures were created by the Chem3D software and exported to mol2 format. Then, the PDB format of the hub protein domain was downloaded from the Protein Data Bank database (https://www.rcsb.org/). The protein was dehydrated and dephosphorized using the PyMOL software, and AutoDockTools1.5 was used. The software was employed to convert the PDB format of active components of drugs and hub gene file to the pdbqt format and search for the active pocket. Finally, the Vina script was run to calculate the molecular binding energy, followed by the display of the molecular docking results. Meanwhile, the Discovery Studio 2019 was run to find the docking sites and calculate the flexible binding LibDockScore. The output molecular docking results were imported into the PyMOL software for the display of molecular docking conformations. If the binding energy was less than 0, the ligand and the receptor could bind spontaneously. When the Vina binding energy was less than −5.0 kcal·mol^−1^ and the LibDockScore was greater than 100, the ligand-receptor complex formed a stable docking. The molecular docking results of the ligand-receptor complex were displayed in 3D and 2D to evaluate the reliability of bioinformatics analyses and predictions.

#### 2.2.8. Molecular Dynamics Simulation

The optimal conformation in the molecular docking was utilized as the initial structure for further molecular dynamics simulations. Based on the docked complex, the all-atomic molecular dynamics simulation was carried out using the classical molecular dynamics simulation software GROMACS (2020.06), analyzing the existing mechanism and verifying the reliability of the binding model. The Amber99SB-ILDN force field parameters were utilized for receptor proteins and ligand molecules, and the ligand molecular topology file was generated using Antechamber and ACPYPE programs. After the dodecahedral solvation box was selected, the nearest distance between the system boundary and the complex was set as 1.5 nm. Then, the TIP3P water model was selected and Na^+^ or Cl^−^ was randomly added to the complex system using the VERLET truncation method to counteract the charge carried by the system. The energy of the system was reduced. NVT was in charge of the system's temperature regulation, which was kept at 300 K. The pressure was controlled by NPT to make the pressure constant at 101.325 kPa. Based on the abovementioned equilibrium, the free kinetic simulation was implemented for 100 ns. The root mean square deviation (RMSD) was adopted to represent the degree of molecular structure changes to measure the stability of the complex system. In the meantime, the root mean square fluctuation (RMSF) and the radius of gyration (Rg) were utilized to analyze the fluctuation of protein structures and folding tightness. The change in the protein binding cavity was reflected by the solvent accessibility surface area (SASA). Subsequent to the analysis of changes in the number of hydrogen bonds between receptor proteins and ligand molecules with simulation time, the receptor-ligand binding free energy was calculated using the molecular mechanics Poisson–Boltzmann surface area (MM-PBSA) method, and the trajectory data were analyzed by using the Molecular Dynamics module.

## 3. Results

### 3.1. Selection of DEGs between Gastric Cancer and Adjacent Normal Tissues

**Figure 1:**
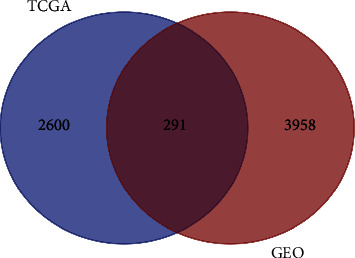
The Venn diagram of co-DEGs in the TCGA and GEO databases. The blue circle showed the DEGs from TCGA database, and the red circle showed the DEGs from GEO database; the co-DEGs were at the overlap part.

The 501 and 3893 statistically significant DEGs obtained from the GSE10323 and GSE54129 datasets, respectively, were merged and de-duplicated to obtain 4249 genes. In addition, 2891 DEGs that conformed to the screening criteria were acquired from the TCGA database. The 2 sets of data were entered into the Draw Venn Diagram for a map analysis, which finally yielded a total of 291 intersecting genes in the GEO and TCGA data, that is, significant gastric cancer-related DEGs (Figure 1).

### 3.2. GO and KEGG Enrichment Analyses of Significant DEGs in Gastric Cancer Tissues versus Adjacent Normal Tissues

**Figure 2:**
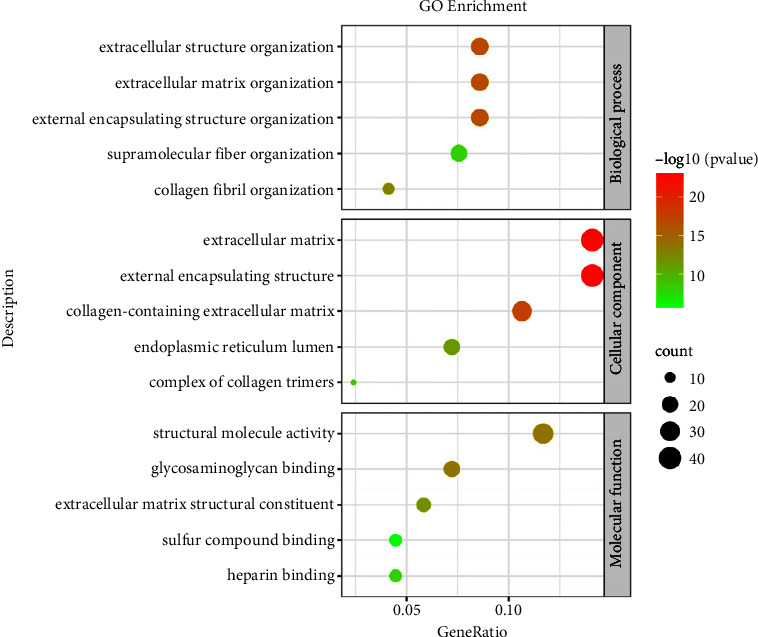
The result of the GO enrichment analysis. The top, middle, and bottom parts symbolized the function of biological process, cellular component, and molecular function, respectively. Different color of spots shows the −log10 (*p* value), and the size of spots means the count of enriched DEGs.

The results of the GO analysis showed that DEGs were mainly involved in an extracellular matrix (ECM), a collagen-containing extracellular matrix, a structural molecule activity, and an external encapsulating structure (Figure 2).

**Figure 3:**
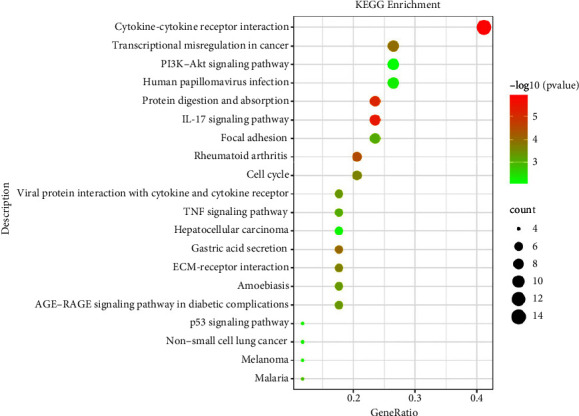
The results of the KEGG pathway enrichment analysis. Different color of spots shows the −log10 (*p* value), and the size of spots means the count of enriched DEGs.

The KEGG analysis results manifested that DEGs were majorly enriched in various signaling pathways, including IL-17 signaling pathway, TNF signaling pathway, protein digestion and absorption, gastric acid secretion, transcriptional misregulation in cancer, ECM-receptor interaction, focal adhesion, PI3K-Akt signaling pathway, cell cycle, and p53 signaling pathway (Figure 3).

### 3.3. Construction of the PPI Network and Screening of Hub Genes

**Figure 4:**
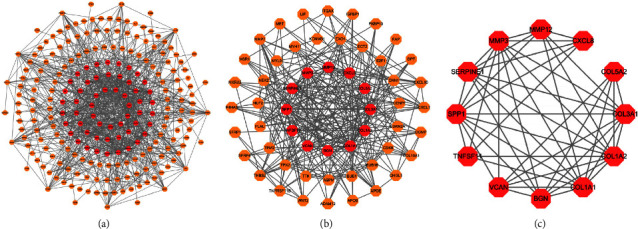
The screening map of hub genes in the PPI network.

The PPI network is demonstrated in Figure 4(a). Twelve network hub genes, including TNFSF11, SERPINE1, COL1A1, matrix metalloproteinase 12 (MMP12), BGN, COL5A2, COL3A1, Versican (VCAN), MMP3, CXCL8, COL1A2, and SPP1, were screened out from the PPI network using the CytoNCA plug-in in the Cytoscape software (Figures 4(b) and 4(c)).

### 3.4. The Survival Analysis and Verification of Hub Genes

**Figure 5:**
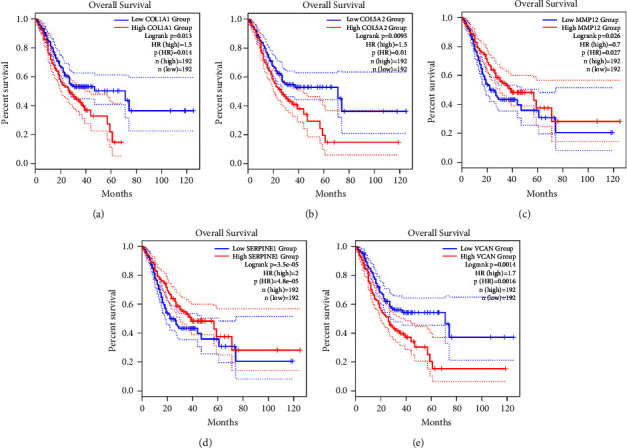
The results of the survival analyses. (a) The survival analysis of SERPINE1 (*P* < 0.05; HR = 1.5). (b) The survival analysis of COL1A1 (*P* < 0.05; HR = 1.5). (c) The survival analysis of MMP12 (*P* < 0.05; HR = 0.7). (d) The survival analysis of COL5A2 (*P* < 0.05; HR = 2). (e) The survival analysis of VCAN (*P* < 0.05; HR = 1.7).

**Figure 6:**
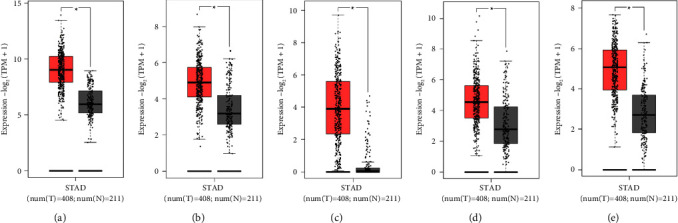
Relative mRNA levels of the five hub genes in gastric cancer and normal gastric tissues. Bexhill showed that (a) COL1A1, (b) COL5A2, (c) MMP12, (d) SERPINE1, and (e) VCAN had elevated mRNA levels in gastric cancer tissues compared with normal gastric tissues. The red color stood for gastric cancer tissues and the grey color marked normal gastric tissues.^*∗*^*P* < 0.05.

The prognostic value of 12 hub genes was scientifically evaluated using the GEPIA database, displaying that five hub genes, SERPINE1, COL1A1, MMP12, COL5A2, and VCAN, exerted an obvious effect on the overall prognosis and survival of patients (Figure 5). In Figure 5, the log-rank*P* < 0.05 represented that the prognosis and survival of patients with high gene expression were statistically different from those of patients with low gene expression. HR stood for the probability of cancer progression or death in patients with high gene expression relative to those with low gene expression. For instance, HR = 1.5 suggested that the risk of cancer progression or death in patients with high gene expression was 1.5 times higher than that in patients with low gene expression. The results documented that the high expression of SERPINE1, COL1A1, COL5A2, and VCAN was associated with poor overall survival (*P* < 0.05; HR > 1), whereas the high expression of MMP12 predicted a favorable prognosis (*P* < 0.05; HR < 1). Furthermore, the differences in the expression of these five genes between gastric cancer and normal gastric tissues were further confirmed in the GEPIA database, which depicted that the mRNA levels of the abovementioned five genes were substantially higher in gastric cancer samples than in normal gastric samples (*P* < 0.05; Figure 6).

### 3.5. Verification of Hub Genes by the Pathology Section Assay

**Figure 7:**
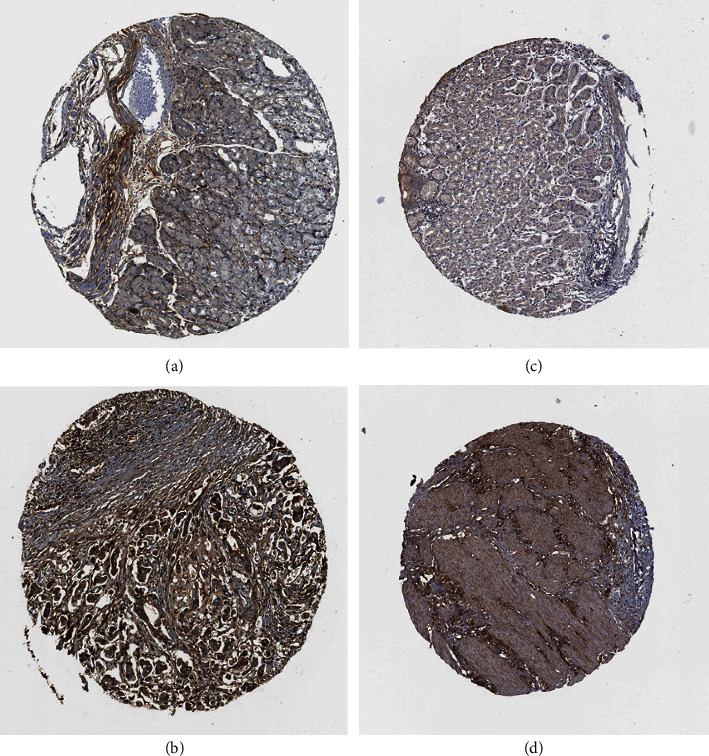
Confirmation of the expression of the hub genes at the translation level based on the HPA database. (a) COL1A1 protein levels in normal tissues (staining, not detected; intensity, weak; quantity, <25%). (b) COL1A1 protein levels in tumor tissues (staining, high; intensity, strong; quantity, >75%). (c) VCAN protein levels in normal tissues (staining, not detected; intensity, weak; quantity, <25%). (d) VCAN protein levels in tumor tissues (staining, high; intensity, strong; quantity, 75%–25%).

In addition to studying the mRNA levels of the hub genes, their protein levels were also measured using immunohistochemistry analysis through the HPA database. Due to the lack of immunohistochemistry staining information for the gastric cancer-related COL5A2, MMP12, and SERPINE1, the representative staining results of COL1A1 and VCAN were selected and are exhibited in Figure 7. There existed more positive expression results of COL1A1 and VCAN in gastric cancer tissues than in normal gastric tissues, indicating elevated protein levels of COL1A1 and VCAN in gastric cancer tissues. These results were concordant with the results of mRNA levels and validated our findings in another way.

### 3.6. Retrieval of Active Ingredients of TCM Targeting Genes Closely Related to Gastric Carcinogenesis

Based on the CTD database, the active compounds of TCM that acted on SERPINE1 comprised quercetin, resveratrol, and emodin. The active compounds of TCM that targeted COL1A1 incorporated quercetin, resveratrol, and schisandrin B (Table 1).

### 3.7. Results of Molecular Docking

**Figure 8:**
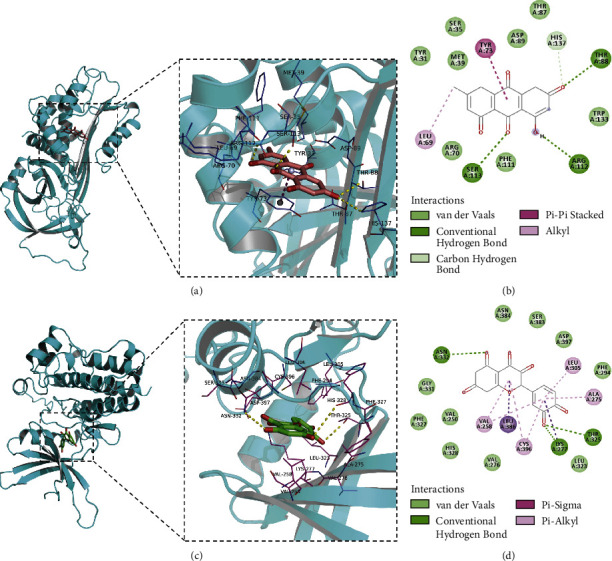
Molecular docking diagram. The features of molecular docking of ERPINE1(3UT3)-Emodin-3D (a), SERPINE1(3UT3)-Emodin-2D (b), COL1A1(7DV6)-Quercetin-3D (c), and COL1A1(7DV6)-Quercetin-2D (d).

The results of the Vina docking showed that the hub proteins (SERPINE1 and COL1A1) could form stable *t*-docking with the corresponding active compounds of TCM, with a binding energy of lower than −5.0 kcal·mol^−1^ (Table 2). In addition, the active ingredients of TCM were docked with corresponding target proteins using the Discovery Studio 2019 software, followed by the calculation of the LibDockScore. The docking sites were observed for all hub proteins (SERPINE1 and COL1A1) and active ingredients of TCM. Among them, the docking models formed by SERPINE1 with quercetin, resveratrol, and emodin and COL1A1 with quercetin all had larger than 100 of LibDockScore, whereas the docking models formed by others possessed less than 100 of LibDockScore. Finally, the compound results output by the Vina was introduced into the PyMOL software, and 3D and 2D molecular docking with protein ligands was displayed using the Discovery Studio 2019 software.  Figure 8 depicts the best combinations of the docking between target proteins and active compounds: SERPINE1 (3UT3)-Emodin and COL1A1 (7DV6)-Quercetin.

### 3.8. Results of Molecular Dynamics Simulations

#### 3.8.1. Results of RMSD

**Figure 9:**
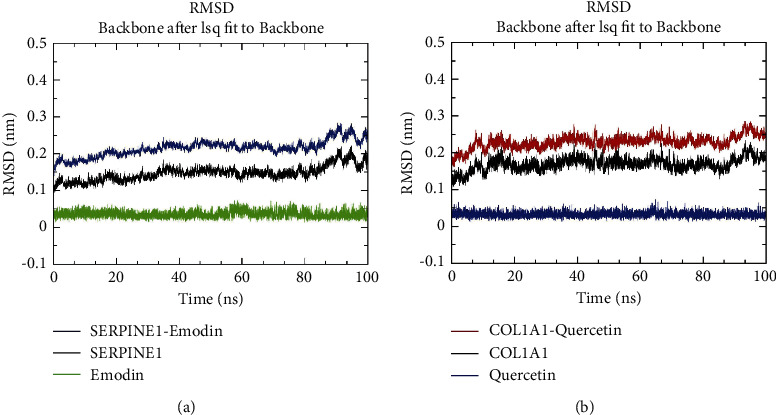
The root mean square deviation. The results of the root mean square deviation of SERPINE1 (a) and COL1A1 (b) complexes.

Combined with the results of the molecular docking, molecular dynamics simulations were performed on the abovementioned conformations as follows: SERPINE1 (3UT3)-Emodin and COL1A1 (7DV6)-Quercetin. RMSD stands for the distance between the same atoms at different simulation times, which can reveal the position changes between the protein conformation and the initial conformation during the simulation process. The changing trend of RMSD of proteins and ligands is also a momentous index to judge whether the simulation is stable or not. The analysis manifested that the SERPINE1-Emodin complex exhibited a certain degree of stability during the simulation, with the mean RMSD of 0.215 nm (max = 0.284 nm, min = 0.102 nm). The value of the system increased slowly within 5–40 ns, and the curve tended to be stable after 40 ns. Meanwhile, it was also noted that there was a peak fluctuation in the RMSD curve of all complexes with SERPINE1 protein after 80 ns, whilst the curve of emodin was still stable. It was speculated that there might exist a certain conformational transformation of the protein at this time (Figure 9(a)), not the disturbance caused by the unstable binding of emodin.

The value of RMSD in the COL1A1-Quercetin complex system was in the range of 0.162–0.286 nm (mean = 0.229 nm). Moreover, the value of RMSD was elevated continuously within 0–20 ns. The curve converged and maintained stably after 20 ns and fluctuated (max = 0.284 nm) after 90 ns, which was also caused by the conformational change of the protein itself. It was worth noting that emodin and quercetin molecules showed a high degree of stability throughout the simulation process, further confirming the reliability and stability of the binding (Figure 9(b)).

#### 3.8.2. Results of RMSF

**Figure 10:**
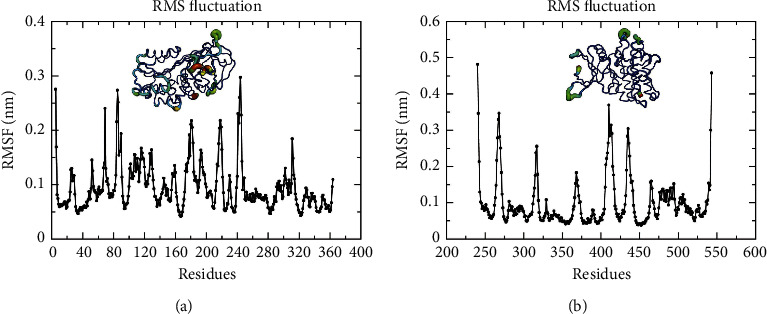
The root mean square fluctuation values of residues. The result of the root mean square fluctuation values of residues in SERPINE1 (a) and COL1A1 (b) complexes.

RMSF is the average atomic position change for the time, which can characterize the flexibility of protein structure and the intensity of motion throughout the simulation. In this study, RMSF values were adopted to ascertain the structural flexibility of protein binding to ligands and the volatility of binding active amino acids. As manifested in Figure 10(a), the SERPINE1 protein contained a variety of flexible regions (max = 0.297 nm) and mainly was the loop structure. In addition, the RMSF values of amino acid residues in other regions were less than 0.2 nm, which demonstrated certain structural stability (mean = 0.095 nm).

As manifested in Figure 10(b), the COL1A1 protein consisted of approximately 5-segment loop structures with obvious volatility, with the highest RMSF value in the free end (max = 0.482 nm). On the other hand, the fluctuation of the residues located in the binding cavity to quercetin (THR325, ALA326, HIS328, and ASN332) was considerably lower than that of the residues in other regions, indicating that the persistent interaction between quercetin and COL1A1 proteins could stabilize the related structures and residues.

#### 3.8.3. Hydrogen Bonds

**Figure 11:**
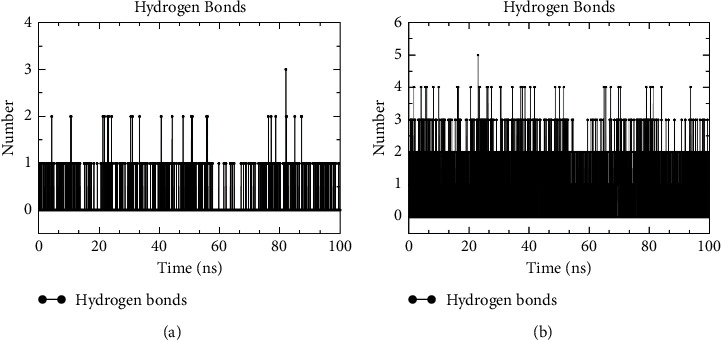
Variation of hydrogen bonds. The result of variation of hydrogen bonds in SERPINE1 (a) and COL1A1 (b) complexes with the simulation time.

Hydrogen bonds assume a key role in the formation and maintenance of the complex and also afflict the stability of ligand-protein binding. The hydrogen bond formation between ligand molecules and receptor proteins was dynamically observed in the time scale of 100 ns dynamics simulation. The results documented that the emodin molecule steadily formed one hydrogen bond with the SERPIINE1 protein (Figure 11(a)). In addition, the COL1A1-Quercetin complex formed two hydrogen bonds on average (Figure 11(b)) and continued to form hydrogen bonds with residues HIS328 and ASN332. A certain number of hydrogen bonds also stabilized the binding conformation of the complex.

#### 3.8.4. Rg

**Figure 12:**
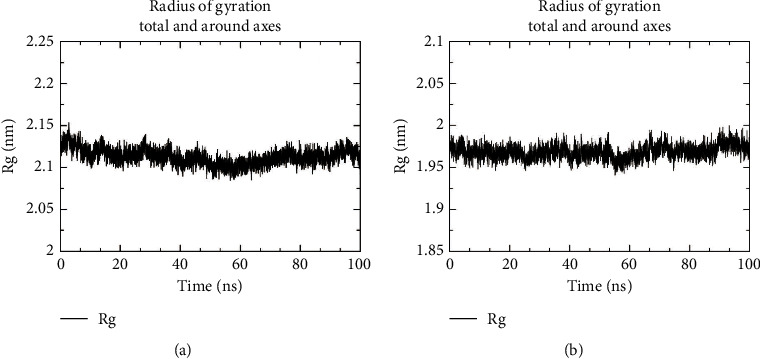
Gyration radius of protein. The result of gyration radius of protein in SERPINE1 (a) and COL1A1 (b) complexes.

Rg can characterize the compactness of protein structure and also reflect changes in a protein-peptide chain looseness during the simulation. This study analyzed the compactness of the structure of SERPINE1 and COL1A1 proteins subsequent to ligand binding and then ascertained whether the ligands depolymerized the protein or impacted the normal folding of proteins. Admittedly, the smaller the Rg value, the more normal and stable the structure. However, this value also is influenced by the structure of the protein itself. Thereby, attention also needed to be paid to the stability of the curve. As described in Figure 12(a), there was no marked change in the conformational folding of SERPINE1 protein binding to the emodin molecule, with the value in the range of 2.129–2.153 nm (mean = 2.113 nm) during the whole simulation. As discovered in Figure 12(b), similar to the SERPINE1 protein, the Rg curve of COL1A1 protein was stable (mean = 1.968 nm) with the value in a small range (max = 2.001 nm, min = 1.941 nm), which also be considered that the protein structure was stable and that the binding of quercetin did not affect the conformation of the COL1A1 protein.

#### 3.8.5. Results of SASA

**Figure 13:**
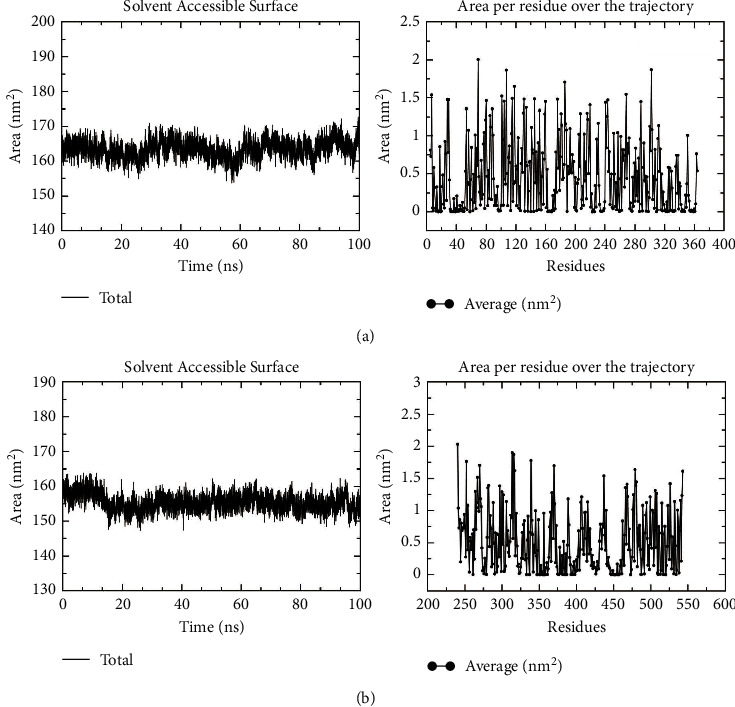
Changes in solvent accessibility and surface area of protein. The result of solvent accessibility and surface area of the SERPINE1 protein (a) and COL1A1 protein (b).

The SASA of SERPINE1 protein is detailed in Figure 13(a), with an average value of 163.399 nm^2^ (max = 172.691 nm^2^, min = 151.246 nm^2^) and periodical changes. Thus, it was speculated that the protein had a conformational transition during 25 ns, but the curve remained highly consistent and stable overall. The correlation of the SERPINE1 protein structure presented that there were several loop structures around the emodin molecules, located binding cavity with certain fluctuations, which influenced the contact between local solvent molecules and proteins. However, the stable conformation of emodin did not signally change the overall SASA value.

The SASA value of COL1A1 protein fluctuated in the range of 147.332–168.371 nm^2^ (mean = 155.392 nm^2^). The SASA value was reduced gradually from the time of 15 ns, reached stable at 17 ns, and maintained this state continuously thereafter (Figure 13(b)). The result reflected that quercetin molecules occupied the protein binding cavity to discharge the existing water molecules inside and thus diminishing its SASA value. The high stability of the curve demonstrated that quercetin bound stably in the cavity and did not present with obvious conformational changes. In addition, when the SASA value was decomposed into each amino acid residue, it was observed that the residues binding to quercetin (THR325, ALA326, HIS328, and ASN332) had lower SASA values, which could also prove the high stability of the abovementioned binding conformation.

#### 3.8.6. Binding Free Energy

**Figure 14:**
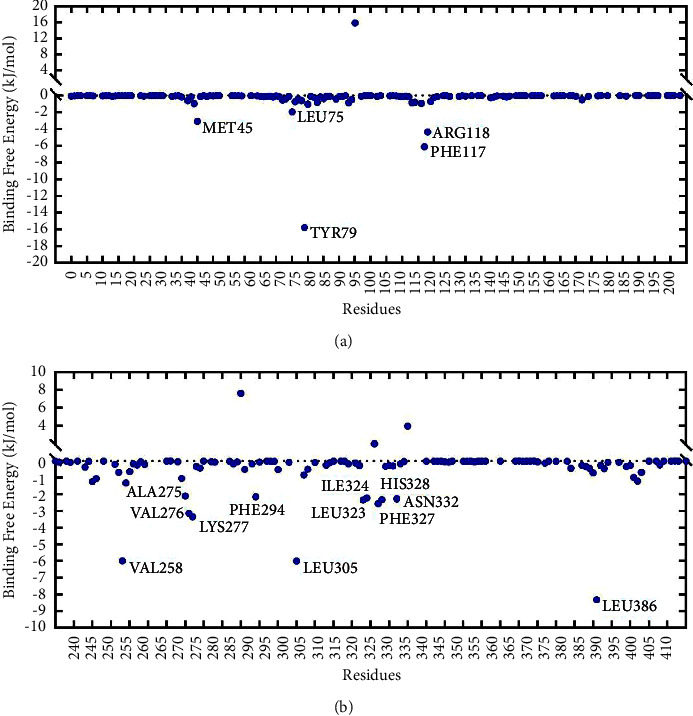
Binding free energy decomposition. The results of binding free energy decomposition in SERPINE1 (a) and COL1A1 (b) complexes.

The binding free energy of the complex in our research was calculated using the widely applied g_mmpbsa script [19]. The results displayed the specific values of each energy as shown in Table 3. Also, the binding strength of ligand molecules to target proteins was quantitatively analyzed by Δ*G*_bind_. The binding free energy was −96.588 kJ/mol for the SERPINE1-Emodin complex. The residue TYR79 (−15.773 kJ/mol) in SERPINE1 protein had the most prominent energy contribution, followed by PHE117 (−6.127 kJ/mol), ARG118 (−4.348 kJ/mol), MET45 (−3.066 kJ/mol), and LEU75 (−1.928 kJ/mol). The hot spot residues that interacted with emodin were distributed around it to ensure the stability of the binding during the simulation (Figure 14(a)). The binding free energy (−114.307 kJ/mol) between COL1A1 protein and quercetin was lower than that of the SERPINE1-Emodin complex. Among hot spot residues, LEU386 (−8.307 kJ/mol), LEU305 (−5.992 kJ/mol), and VAL258 (−5.99 kJ/mol) contributed to outstanding binding free energy and played key parts in maintaining the binding mode of the COL1A1-Quercetin complex (Figure 14(b)).

## 4. Discussion

Despite the advances in therapies for gastric cancer, overall survival and prognosis remain unsatisfactory. In recent years, the rapid development of various bioinformatics technologies provides a viable avenue for the discovery of novel tumor-related diagnostic and therapeutic biomarkers.

In this study, DEGs in gastric cancer were first identified by analyzing gene expression data from TCGA and GEO databases, which acquired a total of 291 intersecting DEGs. The GO enrichment analysis revealed that DEGs were primarily enriched in ECM, collagen-containing extracellular matrix, structural molecule activity, and external encapsulating structure. The KEGG results elucidated that DEGs were predominantly enriched in the ECM-receptor interaction signaling pathway, the PI3K/Akt signaling pathway, the p53 signaling pathway, and so on. The ECM is physiologically essential for intercellular signal transmission, intercellular interaction, and orchestration of cell proliferation, differentiation, and migration [20, 21]. ECM can impede tumor cell migration and invasion, and when its integrity is compromised, tumor cells are more prone to migrate and invade the microenvironment [22]. The PI3K/Akt signaling pathway can manipulate a wide range of biological behaviors of cells, and abnormalities in the PI3K/Akt signaling pathway may trigger the development of gastric cancer [23]. In addition, the p53 signaling pathway is one of the most classic antioncogenic pathways, and p53 transcription factors are implicated in the mediation of numerous transcriptional processes and cellular processes, such as maintenance of genomic stability, cell metabolism, cell apoptosis, cell migration/invasion, and other biological processes [24, 25]. A prior study [26] has found that p53 overexpression dramatically represses the growth and metastasis of tumor cells, which is due to the molecular basis of the excellent anticancer impact of p53 and explains the mutation of the p53 locus in nearly half of cancer patients [24]. In addition, the cell cycle signaling pathway is a fundamental process of cell proliferation, the enhanced activity of which can lead to tumor progression [27]. Of note, the deregulation of the cell cycle signaling pathway is a critical cause of uncontrolled cell proliferation [28]. In summary, massive research has unraveled that the ECM, PI3K/Akt, p53, and cell cycle signaling pathways are tightly associated with the development of gastric cancer, indicating the reliability of the bioinformatics analysis results in this study for the regulatory network of gastric cancer pathogenesis.

Our study predicted 12 hub genes with a strong correlation with gastric carcinogenesis. Moreover, combined with the prognostic value, it was observed that the alterations of five genes, COL1A1, COL5A2, MMP12, SERPINE1, and VCAN, were strongly related to the poor overall survival of patients. COL1A1 and COL5A2 both belong to the collagen family, which is a major component of the ECM [29]. More importantly, the upregulation of collagens assumes a critical role in the promotion of tumor growth. COL1A1, as the most abundant protein in the encoded collagen family, is a primary component of the ECM that can afflict cell behaviors and tissue structures [30]. Li et al. [31] concluded that COL1A1 suppressed proliferation, migration, and invasion of gastric cancer cells. In addition, Zhang et al. [32] further demonstrated that ectopic COL1A1 facilitated gastric cancer cell proliferation *in vitro*. A mechanistic study elucidated that COL5A2 might accelerate tumor progression through hypoxia, coagulation, apical junction, angiogenesis, and apoptosis [33]. Tan et al. [34] discovered that COL5A2 upregulation contributed to the facilitation of gastric cancer cell migration. Together, these studies illuminate that COL1A1 and COL5A2 may not only be reliable biomarkers of gastric cancer cell proliferation and metastasis, and also key predictors of poor prognosis in patients with gastric cancer.

The dysregulation of MMP12 (also known as human macrophage metalloelastase) has been hypothesized to be linked to all sorts of cancers, such as gastric cancer [35], but it predicts different prognoses in different tissues. Cheng et al. [36] found that the high expression of MMP12 predicted a good prognosis in gastric cancer due to a tight correlation with reduced angiogenesis and vascular infiltration, which could function as a valid predictor for patients with gastric cancer. Consistently, the present study also elaborated that gastric cancer patients with MMP12 high expression had longer overall survival.

A prior study [37] reported that the SERPINE1 gene, also termed PAI-1, was associated with oncogene activation. Another mechanistic study unraveled that SERPINE1 enhanced metastasis in gastric cancer and accelerated peritoneal tumor growth in a mouse model of gastric cancer metastasis [38]. Also, it was clarified in previous research [39] that SERPINE1 was a potent biomarker correlated with epithelial-mesenchymal transition in gastric cancer. The research of Yang et al. [40] identified that SERPINE1 could promote tumor cell proliferation, migration, and invasion by manipulating EMT and that SERPINE1 overexpression culminated in a poorer prognosis and could be an independent prognostic factor for patients with gastric adenocarcinoma.

VCAN, a multifunctional proteoglycan, is a member of the proteoglycan family, which is a main component of the ECM. It has been documented that VCAN is aberrantly expressed in a huge range of tumors, such as breast [41], ovarian [42], and colorectal [43] tumors and plays a pivotal role in tumor cell invasion, metastasis, and immune infiltration. Accumulating research [44, 45] unveiled that VCAN is highly expressed in gastric cancer and closely related to the survival of patients with gastric cancer patients, which thereby might act as an essential prognostic marker for the survival of patients with gastric cancer. Huang et al. [46] suggested that VCAN might impact the development of gastric cancer by modulating the tumor microenvironment, which might be a potential therapeutic target for gastric cancer. The prediction in our research is concurrent with the abovementioned findings and illustrated that VCAN may represent a novel prognostic biomarker for gastric cancer.

The search for target genes is a very important part of the drug discovery process. Intriguingly, mounting molecules, compounds, and drugs have been noted to share complex relationships with a large quantity of genes and proteins [47–49]. TCM and natural compounds contain a large number of active ingredients, which provides more possibilities and opportunities for drug development and use. In this study, a total of four potential active ingredients in TCM were identified based on the CTD database, including quercetin, resveratrol, emodin, and schisandrin B, all of which could be utilized in the prevention and treatment of gastric cancer.

It is extensively accepted that Vina binding energy to the receptor protein < −5.0 kcal·mol^−1^ or LibDockScore > 100 indicates the strong binding power of compounds. Our data displayed that all of the four screened active ingredients of TCM had less than −5.0 kcal·mol^−1^ of binding to the corresponding protein receptor molecules in the docking results. At the molecular level, the aforesaid results illustrated that the potential therapeutic effects of these four active ingredients, especially the SERPINE1-Emodin complex and the COL1A1-Quercetin complex, for gastric cancer not only possessed Vina binding energy < −5.0 kcal·mol^−1^ but also had LibDockScore > 100. Molecular dynamics simulations help unveil various dynamic interactions between a ligand and receptor, their interaction mechanism, and stability [50]. Here, we found that SERPINE1-Emodin and COL1A1-Quercetin could demonstrate relatively stable binding, which was consistent with the molecular docking results. Of note, Van der Waals potentials, electrostatic, and hydrogen bonding are most critical for their stable binding. SERPINE1 and COL1A1 have high overall flexibility and contain multiple flexible regions, which may be related to the specific structure of the proteins. Based on this, sufficient attention should be paid to the research of the mechanism of these two complexes in the improvement or the overall pathogenesis and treatment of gastric cancer.

Quercetin is a common flavonoid that is an active ingredient in numerous Chinese herbal medicines. Quercetin has been reported to exhibit antioxidant [51], anti-inflammatory [52, 53], and antimicrobial activities [54, 55] and is also considered an anticancer agent [56]. A large body of epidemiological evidence has elucidated that the consumption of quercetin-rich vegetables and fruits may prevent the development of several cancers [57, 58]. Quercetin exerts antitumor impacts on gastric cancer cells by inducing apoptosis [59]. Another study [60] manifested that Quercetin could mediate the Akt-mTOR and hypoxia-induced factor 1 *α* (HIF-1*α*) signaling pathways to activate the autophagic process in gastric cancer cells and also restrict gastric cancer cell metastasis by blocking the uPA/uPAR function [61].

Emodin, the principal active ingredient of the Chinese herbal medicines, *Rheum officinale*, *Polygonum multiflorum*, and Aloe leaves, is an anthraquinone derivative with various pharmacological activities, including antioxidant, anticancer, and anti-inflammatory effects [62]. Notably, prior research [63] has unraveled that emodin can subdue cell proliferation, facilitate cell apoptosis, and alter cell redox status, invasion, metastasis, and tumor angiogenesis. Currently, emodin has been evidenced to impede the growth of cells in lung, colon, and gastric cancers [64, 65].

## 5. Conclusion

Altogether, we analyzed the prognostic value of the hub genes and elucidated the interactions between the genes in gastric cancer pathogenesis. The functional analysis revealed the enrichment of ECM-receptor interaction, PI3K/Akt and p53 in gastric cancer by bioinformatics. Besides, we further mined the active ingredients of TCM targeting the hub genes, and it is shown that bioinformatics combined with molecular docking and molecular dynamics simulations can not only screen the hub pathogenic genes and potential active ingredients of medicines but also unveil the binding pattern of small-molecule ligands to protein receptors of the disease. We hope this study could provide a novel perspective for the biomarkers screen and TCM active ingredients' selection in gastric cancer.

## Figures and Tables

**Table 1:** Active ingredients of TCM targeting the hub pathogenic genes of gastric cancer.

Genes	Active ingredients of TCM	ID of ingredient	Interaction number
SERPINE1	Quercetin	D011794	4
	Resveratrol	D000077185	9
	Emodin	D00442	3

COL1A1	Quercetin	D011794	6
	Resveratrol	D000077185	16
	Schisandrin B	C015499	4

**Table 2:** Results of molecular docking using Vina and Discovery Studio 2019.

Structural domains	Compounds	Vina (kcal·mol^−1^)	RMSD	DS (LibDockScore)	Hydrogen bond interaction	Hydrophobic interaction
SERPINE1 (3UT3)	Quercetin (D011794)	−7.9	0.631	114.8	THR: 215, GLU: 283	THR: 282.LYS: 323, LEU: 152
SERPINE1 (3UT3)	Resveratrol (D000077185)	−7.2	0.463	104	—	PRO: 289, ALA: 26, LYS: 288, VAL: 23, VAL: 32, VAL: 284
SERPINE1 (3UT3)	Emodin (D00442)	−8.9	1.214	115.1	SER: 113, ARG: 112, THR: 88; HIS: 137	ASP: 39, Tyr: 73; Phe: 111
COL1A1 (7DV6)	Quercetin (D011794)	−9.9	1.331	107.9	ASN: 332, HIS: 328, THR: 325, LYS: 277	VAL: 258, LEU: 386, ALA: 275, LEU: 305, CYS: 396
COL1A1 (7DV6)	Resveratrol (D000077185)	−8.3	1.387	96.48	HIS: 328, LYS: 277, LEU: 323, THR: 325	PHE: 327, LEU: 386, ALA: 275, CYS: 396, LEU: 305, VAL: 258, GLU: 290
COL1A1 (7DV6)	Schizandrin B (C015499)	−6.0	2.372	98	ARG: 254, ALA: 256, VAL: 250	ASP: 397

**Table 3:** MM-PBSA based total binding free energies along with its constituent energies for the complexes.

Complexes	Total binding free energy (kJ/mol)	Van der Waals energy (kJ/mol)	Electrostatic energy (kJ/mol)	Polar solvation energy (kJ/mol)	SASA energy (kJ/mol)
SERPINE1-emodin	−96.588	−149.932	−15.255	79.339	−17.344
COL1A1-quercetin	−114.307	−188.906	−4.450	80.704	−19.392

## Data Availability

The datasets generated during and/or analysed during the current study are available from the corresponding author on reasonable request.
